# Experiments on the Pancreatic Fluid of the Calf

**Published:** 1851-07

**Authors:** J. L. Lassaigne


*Experiments on the Pancreatic Fluid of the Calf.*—By M. J. L. Lassaigne.—Since it has been shown that the pancreatic fluid extracted from the dog, enjoys the remarkable property of making an emulsion with oil, and of causing its transformation into fatty acids, either at ordinary temperatures or at the temperatures of the bodies of mammalia; it has become matter of interest to submit to similar experiments the pancreatic fluid obtained from a large herbivorous animal, with the view of ascertaining if it acts in the same manner.

M. Colin, director of the anatomical department of the school of Alfort, having procured some of it, by the method shown to him by M. Bernard, and which he had successfully put in practice with the dog, a small quantity was sent to M. Lassaigne, which he immediately examined.

The pancreatic fluid of the calf, which has an amber yellow colour, is inodorous, is transparent and limpid, and possesses a tolerably marked saline taste; when tested with reddened litmus paper, it restores the blue tint. Heated, it becomes opalescent and turbid, and deposits some coagulated white flakes, which attach themselves to the sides of the vessel in which the experiment is made.

The small quantity at the author's disposal not permitting of a direct analysis, he thought it right to examine, in concert with M. Colin, the action which it exercises on oils.

For this purpose he divided it into several portions in glass tubes, and added to each portion a drop of pure olive oil. The agitation to which he subjected the tubes, formed no emulsion—the oil separating speedily, and floating on the top.

The prolongation of the experiment did not produce any effect, even after eight hours of contact at a temperature of 60 degrees Fahr., the oil having remained neutral, and the liquid preserving its primitive alkalinity. Subsequently exposing these same tubes in a salt-water bath to 100 degrees Fahr., and keeping up this temperature for fourteen hours, produced no change in the properties of the oil, or in those of the pancreatic juice subjected to the experiment.

This result is opposed to that obtained from the pancreatic juice of the dog,—is it constant in the herbivorous animal, or is it an exceptional occurrence due to an alteration in the pancreatic juice of the animal, caused by the painful operation to which it had been subjected? The author does not admit the latter hypothesis, seeing that the animal survived, and did not appear to suffer in health.—*Lond. Monthly Journal of Med. Sience. Journ. de Pharm.*, March, 1851.

